# Validation of a digital identification tool for individuals at risk for hereditary cancer syndromes

**DOI:** 10.1186/s13053-018-0099-8

**Published:** 2019-01-11

**Authors:** Leslie Bucheit, Katherine Johansen Taber, Kaylene Ready

**Affiliations:** Counsyl, 180 Kimball Way, South San Francisco, CA 98040 USA

**Keywords:** BRCA1, BRCA2, Hereditary breast and ovarian Cancer, Lynch syndrome, Hereditary cancer, Family history screening. Risk assessment, Digital tool, Health information technology

## Abstract

**Background:**

The number of individuals meeting criteria for genetic counseling and testing for hereditary cancer syndromes (HCS) is far less than the number that actually receive it. To facilitate identification of patients at risk for HCS, Counsyl developed a digital identification tool (digital ID tool) to match personal and family cancer history to National Comprehensive Cancer Network (NCCN) BRCA-related Hereditary Breast and Ovarian Cancer (HBOC), Lynch syndrome, and polyposis testing criteria in one-to-one, automated fashion. The purpose of this study was to validate the ability of the digital ID tool to accurately identify histories that do and do not meet NCCN testing criteria.

**Methods:**

Third-party recorded three-generation pedigrees were retrospectively reviewed by a certified genetic counselor (CGC) to determine if independent events included in pedigree histories met NCCN guidelines, and were then sorted into groups: high risk events (meets criteria) and low risk events (does not meet criteria). Events were entered into the digital ID tool to determine the extent of its concordance with events sorted by CGC review. Statistical tests of accuracy were calculated at a 95% confidence interval (CI).

**Results:**

One hundred ninety-seven pedigrees were reviewed consecutively representing 765 independent events for analysis across groups. 382/382 (100%) high risk events identified by the digital ID tool and 381/383 (99.47%) low risk events identified by the digital ID tool were concordant with CGC sorting. The digital ID tool had a sensitivity of 100% (99.04–100% CI) and specificity of 99.48% (98.13–99.94% CI). The overall accuracy of the digital ID tool was estimated to be 99.74% (99.06–99.97% CI), reflecting the rate at which the digital ID tool reached the same conclusion as that of CGC review of pedigree events for the recommendation of genetic testing for individuals at risk for HCS.

**Conclusions:**

The digital ID tool accurately matches NCCN criteria in one-to-one fashion to identify at-risk individuals for HCS and may be useful in clinical practice, specifically for BRCA-related HBOC and Lynch Syndrome.

**Electronic supplementary material:**

The online version of this article (10.1186/s13053-018-0099-8) contains supplementary material, which is available to authorized users.

## Background

Approximately 5–10% of all cancers are due to hereditary cancer syndromes (HCS) [1]. Select syndromes are relatively prevalent genetic conditions, with mutations in the *BRCA1* and *BRCA2* genes and Lynch syndrome-related genes occurring in approximately 1 in 500 [2] and 1 in 370 [3] individuals, respectively. Germline genetic testing analyzes such genes to help explain personal and/or family history of cancer, determine future cancer risks, and inform medical management recommendations [1, 3–5].

The National Comprehensive Cancer Network (NCCN) publishes criteria to guide identification of at-risk individuals for HCS [4, 5], including well-defined criteria on BRCA-related Hereditary Breast and Ovarian Cancer (HBOC), Lynch syndrome, and polyposis syndromes. Although guidelines are well-referenced and used in clinical practice, 70–80% of women with a personal history of breast or ovarian cancer who meet NCCN criteria report never discussing genetic testing with their health care provider [6, 7] and less than one-third of individuals at risk for Lynch syndrome have been advised by their health care provider to undergo genetic counseling [8]. Even fewer individuals without a personal history of cancer who may be at risk for such conditions report discussing genetic testing or being referred to a specialist to do so [9].

Additionally, individuals at high risk for hereditary cancer syndromes often do not receive genetic testing recommended by guidelines [6, 8–11]. Fewer than 1 in 5 women with a history of breast or ovarian cancer meeting select NCCN criteria have had genetic testing [9]. Among individuals at high risk to harbor a *BRCA1* or *BRCA2* mutation, fewer than 10% report undergoing BRCA-related testing [8]. Similar trends have been observed in the identification of at-risk Lynch syndrome carriers, with fewer than 7% completing genetic testing to clarify their risk status [7]. Across unaffected populations, fewer than 5% of *BRCA1* or *BRCA2* carriers and fewer than 1% of Lynch syndrome carriers are estimated to have been identified through genetic testing [10].

While health care providers recognize the increased impact of genetics on medicine in the last 5–10 years, many never refer patients to genetic counselors or specialists for genetic counseling and/or testing [12]. Such gaps in the uptake of genetic counseling and/or testing may reflect a lack of knowledge about how to identify at-risk patients [6]. The collection of family history as recorded by the gold-standard of clinical practice—a three-generation pedigree—is an essential component of identifying patients at risk for HCS [11, 13]. However, among providers reporting that they routinely assess hereditary cancer risk, only one-third record a full three-generation pedigree [14]. Although NCCN encourages creation of three-generation pedigrees for family history collection [4, 5], more concise family history collection methods might also allow for effective identification of at-risk individuals [11].

Given the low rate of family history collection and genetic counseling and/or testing in high risk populations, alternative tools are needed to better identify patients at risk for HCS in order to trigger appropriate downstream care. A number of validated screening tools have been created based on NCCN guidelines to ascertain at-risk individuals in various practice settings [15, 16]. Questionnaires, web-based tools and other screening methods are valid, sensitive, and are considered as high-quality and acceptable among patients and providers as methods of collecting cancer family history [17–20], although actual use of such tools in clinical practice remain to be evaluated. Alternative tools that collect family history prior to a visit with a health care provider have also been shown to reduce consultation time and allow providers to focus on assessment of a history rather than collection of the history itself [11, 19].

Technological advances and mobile accessibility may also enable automated family history collection using digital tools and mobile devices. We developed a digital, automated patient identification tool (“digital ID tool”) that utilizes a web-based family history assessment optimized for rapid use on a mobile device, and report on its validation here. The digital ID tool uses a link customized to a health care provider’s practice to allow for family history collection prior to or during a patient’s visit. The digital ID tool automatically assesses the individual’s personal and/or family cancer history against NCCN guidelines with the goal of identifying individuals at-risk for common HCS. The digital ID tool outputs reports to health care providers indicating whether an individual’s personal and/or family cancer history meets NCCN criteria for BRCA-related HBOC, Lynch syndrome, or polyposis syndromes [4, 5]. Unlike screening tools developed to determine fulfillment of NCCN criteria based on broader questions and/or self-developed criterion [16, 17, 21], the digital ID tool directly aligns reported cancer histories to NCCN criteria in one-to-one fashion. The accuracy of tools with this one-to-one alignment feature remains to be tested.

Research has shown optimism for adoption of digital technology in the clinical setting [18, 20, 22–24], yet successful implementation requires a reliable, valid tool. This study aims to determine if the digital ID tool can 1) accurately identify at-risk individuals for BRCA-related HBOC, Lynch syndrome, and polyposis conditions utilizing automated alignment of personal and family cancer history to NCCN criteria; and 2) make the same recommendation for or against genetic testing as would be recommended by review of a three-generation pedigree by a clinical genetics expert.

## Methods

To determine if the digital ID tool could accurately identify at-risk individuals who meet NCCN criteria, and to define its sensitivity and specificity, a board certified genetic counselor (CGC) reviewed three-generation pedigrees against NCCN criteria and compared the output review (i.e., meets criteria or does not meet criteria) to the output of the digital ID tool to assess for concordance.

### Obtainment of participant pedigrees

A subset of United States medical insurance policies require pre-test genetic counseling consultation with a third-party, non-laboratory-based CGC as part of the pre-authorization process for coverage of hereditary cancer testing. During pre-test counseling, a three-generation pedigree is compiled to ensure that ordered testing is appropriate and medically indicated. Collection of family histories and generation of pedigrees may occur prior to or after sample receipt by a testing laboratory, but must be completed before the sample is processed and testing begins. These pedigrees reside in the records of the testing laboratory for billing purposes.

Three-generation pedigrees were retrospectively queried from Counsyl’s billing system for patients who received genetic testing for HCS in 2017 and were covered by an insurance policy with a pre-test genetic counseling requirement that included third-party family history collection. Pedigrees were randomly selected for review. The proband’s age at the time of pedigree generation, sex, personal cancer history and ethnicity were recorded into a secure study database. Participants were excluded if their records did not include a three-generation pedigree, if they were under 18 years of age at the time of pedigree recording, or if they had opted out of their data being used for research.

### Pedigree review and group designation

Pedigrees were reviewed by a CGC with experience in providing direct patient care in cancer genetics to determine if the participant’s personal and/or family cancer history met NCCN genetic testing criteria for BRCA-related HBOC, Lynch syndrome, or polyposis syndromes. Queried pedigrees were not pre-selected as meeting NCCN criteria; rather, they were identified as high risk (meets NCCN criteria) or low risk (does not meet NCCN criteria) at the time of CGC review.

The following versions of NCCN guidelines were used in this study for pedigree review: NCCN Clinical Practice in Oncology: Genetic/Familial High-Risk Assessment: Breast and Ovarian V2.2017 and NCCN Clinical Practice in Oncology: Genetic/Familial High-Risk Assessment: colorectal V2.2017. These guidelines include 25 potential criteria for BRCA-related testing, 16 criteria for Lynch syndrome testing and three criteria for polyposis testing. Each criterion was analyzed as an independent event in pedigree review; that is, one pedigree could have fulfilled multiple criterion events.

To establish a high risk group to test the digital ID tool’s ability to accurately identify at-risk histories that meet NCCN criteria (sensitivity), pedigrees were randomly selected from billing records and reviewed consecutively until at least 380 independent events fulfilling NCCN criteria were recorded, based on sample size calculations (see below). Each independent event meeting NCCN criteria during CGC review was recorded and referenced as “meets criteria.” If an event fulfilled NCCN criteria, the output from the digital ID tool was “genetic screening should be considered at this time,” indicating the need for follow-up testing for HCS. If the pedigree was not identified as meeting NCCN criteria during initial review, it was flagged and later analyzed as part of the low risk group.

To test the digital ID tool’s ability to accurately identify low risk histories that do not meet NCCN criteria (specificity), a low risk group was established using cases that did not meet criteria from consecutive review to identify high risk cases as well as modeled cases created from three-generation pedigrees included in the high risk group. Modeled cases were needed because individuals who do not have apparent risk factors for genetic testing often do not seek testing, limiting the dataset available for analysis in this study. To create modeled histories, qualifying events that would otherwise fulfill NCCN criteria were removed from queried pedigrees from the high risk group. All remaining personal and/or family cancer history was then recorded as independent events, including cancer type(s), age of diagnosis as recorded on the pedigree, and degree of relation to the proband. If more than one event occurred within the same lineage, they were also entered as a combination event into the digital ID tool. If no personal or family history existed after removing qualifying events that fulfilled NCCN criteria, pedigrees were analyzed as “no family history” and still qualified for entry into the digital ID tool.

### Assumptions

As is the case with most pedigrees, relatives’ age of diagnosis of cancer were often designated as ranges of decades, early decades, or late decades. However, the digital ID tool does not allow for generalized age input. Therefore, if a pedigree referenced an early decade age, an age with an integer of 2 was entered (e.g., “early 70s” was reviewed and entered as “72”). Similarly, if the pedigree referenced a late decade age, an age with an integer of 8 was entered (e.g., “late 70s” was reviewed and entered as “78”). Finally, if the pedigree referenced an entire decade, the median age of the referenced decade was entered (e.g., “70s” was reviewed and entered as “75”). This method was employed across groups and events analyzed by CGC pedigree review and digital ID tool entry.

Cancer types were accounted for as recorded on the pedigree. For entry into the digital ID tool, if cancer types were not included as a listed option, they were recorded as an “other / unspecified” cancer type. Prostate cancers were assumed to be high-risk (Gleason score > 7) for inclusion, as pedigrees rarely indicate aggressivity of prostate cancer. Pathology records were not included in review and therefore NCCN criteria related to BRCA-related somatic test results and Lynch syndrome-related tumor testing were not tested as part of this study. Risk models, such as PREMM-1, 2, 6 or Tyrer-Cuzick, provide the probability of a mutation being present through calculations and/or Bayes theorem models [25–27], which differ in purpose from the digital ID tool. Therefore, such risk calculations were not included in this study and related NCCN criteria were not tested.

### Digital ID tool development

The digital ID tool was created by a multidisciplinary team at Counsyl, including certified genetic counselors, software engineers, user experience professionals, and product management experts to align inputted histories in one-to-one fashion with NCCN testing criteria for BRCA-related HBOC, Lynch syndrome, and polyposis conditions. The digital ID tool collects personal and family cancer history only and does not include other disease states. Table 1 describes the questions included in the digital ID tool, which uses stepwise logic dependent on respondent answers to optimize usability; the digital ID tool also asks for the number of total relatives on each side of the family and is capable of generating a pedigree report for provider use. Screenshots of the digital ID tool as it would be encountered on a mobile device are included as Additional file 1: Figure S1.

**Table 1:** Categories of questions, prompts included in digital id tool

Primary Question / Prompt	Follow-up Question (where applicable)
1. How would you describe your ethnic background?	*Select appropriate background(s).*
2. Have you ever been diagnosed with cancer?	*If so, what type and at what age?*
3. Is there a family history of cancer?	*If so, who, what type and at what age?*
4. Have you/family member had any colon polyps?	*If so, who/how many?*
5. Has anyone in your family tested positive for a cancer-related gene mutation?	*If so, from BRCA1/BRCA2, Lynch syndrome, APC/MUTYH?*
6. Help us understand the size of your family.	*Enter number of family members in each generation.*

For each cancer history entered into the digital ID tool, the degree of relation of affected relative, cancer type, age of diagnosis, and lineage (where applicable) must be entered. The digital ID tool can record cancer history for personal history and first-, second-, and third-degree relatives, and includes 36 unique types of cancer. For this study, the same NCCN guideline versions included in the digital ID tool were used in pedigree review (NCCN Clinical Practice in Oncology: Genetic/Familial High-Risk Assessment: Breast and Ovarian V2.2017 and NCCN Clinical Practice in Oncology: Genetic/Familial High-Risk Assessment: Colorectal V2.2017). Of note, the digital ID tool was independently reviewed and tested by NCCN staff and certified genetic counselors, and was approved by the NCCN as accurately using NCCN guidelines.

### Sample size, statistics

A sample size of 380 independent events was calculated to provide 90% power to detect a 5% difference between histories meeting NCCN criteria by CGC pedigree review and those meeting the same criteria by the digital ID tool. Therefore, at least 380 independent events were recorded for both the high risk and low risk groups across consecutively reviewed pedigrees. All statistical analysis, including sample size calculation, sensitivity, specificity, and accuracy, were calculated at a 95% confidence interval (CI; assumed type I error rate of 5%).

For accuracy calculations, “sensitivity” was defined as the digital ID tool identifying high risk patients as would a CGC-analyzed pedigree (i.e., correctly identifying at-risk histories as meeting NCCN criteria) and “specificity” was defined as the digital ID tool identifying low risk patients as would a CGC-analyzed pedigree (i.e., correctly identifying low risk histories as not meeting NCCN criteria). True positive and false negative rates were calculated from the high risk group, with “true positive” representing an event fulfilling NCCN criteria on CGC pedigree review and also on the digital ID tool, and false negatives representing the digital ID tool misidentifying a high risk event as low risk. True negative and false positives rates were calculated from the low risk group, with “true negative” representing an event not fulfilling NCCN criteria on pedigree review nor on the digital ID tool, and false positives representing the digital ID tool misidentifying a low risk event as fulfilling NCCN criteria.

## Results

One hundred ninety-seven pedigrees were reviewed consecutively representing 765 independent events for analysis across groups, equating to an event rate of approximately 3.88 events per pedigree. Probands of reviewed pedigrees were all female and largely did not report a personal history of cancer (Table 2). Ethnicity, age distributions, and personal cancer histories differed slightly between high risk and low risk groups (Table 2).

**Table 2:** Demographics and personal cancer history across groups

	High Risk Group	Low Risk Group
Number of probands	197	123
Average age of probands	42.80 years (range 19–75)	44.85 years (range 29–68)
Sex of probands *(N, % of group)*		
Female	197 (100)	123 (100)
Male	0 (0)	0 (0)
Ethnicity *(N, % of group)*		
African American	11 (5.58)	6 (4.88)
Ashkenazi Jewish	17 (8.63)	7 (5.69)
Asian	7 (3.55)	6 (4.88)
Caucasian	145 (73.60)	95 (77.24)
Hispanic	9 (4.57)	6 (4.88)
Native American	5 (2.54)	2 (1.63)
Unknown or Not Reported	3 (1.52)	1 (0.81)
Personal cancer history *(N, % of group)*		
Number of Probands with Personal History	55 (27.92)	18 (14.63%)
Number of Tumors	58 (N/A)	18 (N/A)
Personal history: cancer type *(N, % of tumors)*		
Breast (Total)	42 (72.41)	3 (16.67)
*Breast (Unspecified)*	*37 (63.79)*	*3 (16.67)*
*Breast – DCIS*	*2 (3.45)*	*0 (0)*
*Breast – Triple Negative*	*3 (5.17)*	*0 (0)*
Cervical	2 (3.45)	3 (16.67)
Fallopian Tube Cancer	1 (1.72)	0
Melanoma	3 (5.17)	4 (22.22)
Non-Hodgkin’s Lymphoma	2 (3.45)	1 (5.56)
Ovarian	4 (6.90)	0 (0)
Skin (unspecified)	2 (3.45)	3 (16.67)
Thyroid	1 (1.72)	3 (16.67
Uterine	0 (0)	1 (5.56)
Wilms’ Tumor	1 (1.72)	0 (0)

### High risk group

One hundred ninety-seven pedigrees were included in the high risk group, with 382 independent events recorded to assess against NCCN criteria in CGC pedigree review and for entry into the digital ID tool, representing an event rate of approximately 1.94 events per pedigree (Table 3).

**Table 3:** NCCN criteria tested in high risk group events

BRCA-Related NCCN Criteria (V2.2017)^a^ Fulfilled	Affected^g^	Unaffected^h^	Total Across Events
(*n* = 343)	n (% of BRCA met)		*N* = 382 (%)
Individual from a family with a known BRCA1/BRCA2 mutation	2 (0.58)	10 (2.92)	12 (3.14)
Personal history of breast cancer^b^ and > 1 of the following:			
Diagnosed < 45 y	18 (5.25)	59 (17.20)	77 (20.15)
Diagnosed < 50 y with:			
An additional breast cancer primary	3 (0.87)	6 (1.75)	9 (2.36)
> 1 close blood relative^c^ with breast cancer	11 (3.21)	24 (7.00)	35 (9.16)
> 1 close blood relative with pancreatic cancer	1 (0.29)	1 (0.29)	2 (0.52)
> 1 close blood relative with prostate cancer	3 (0.87)	2 (0.58)	5 (1.30)
An unknown or limited family history	0 (0)	0 (0)	0 (0)
Diagnosed < 60 with triple negative breast cancer	2 (0.58)	3 (0.87)	5 (1.30)
Diagnosed at any age with:			
> 2 close blood relatives with breast cancer, pancreatic cancer, or prostate cancer at any age	12 (3.50)	34 (9.91)	46 (12.04)
> 1 close blood relative with breast cancer diagnosed < 50 y	3 (0.87)	3 (0.87)	6 (1.57)
> 1 close blood relative with ovarian carcinoma	7 (2.04)	23 (6.71)	30 (7.85)
A close male blood relative with breast cancer	0 (0)	1 (0.29)	1 (0.26)
Individual of ethnicity associated with higher mutation frequency (e.g., Ashkenazi Jewish) no additional family history may be required	3 (0.87)	9 (2.62)	12 (3.14)
Personal history of ovarian^d^ cancer	5 (1.46)	75 (21.87)	80 (20.94)
Personal history of male breast cancer	0 (0)	1 (0)	1 (0.26)
Personal history of prostate cancer at any age with > 1 close blood relative with ovarian carcinoma at any age or breast cancer < 50 y or two relatives with breast, pancreatic, or prostate cancer at any age	0 (0)	8 (2.33)	8 (2.09)
Personal history of pancreatic cancer at any age with > 1 close blood relative with ovarian carcinoma at any age or breast cancer < 50 y or two relatives with breast, pancreatic, or prostate cancer at any age	0 (0)	11 (3.21)	11 (2.87)
Family history only:			
First- or second- degree blood relative meeting any of the above criteria (^f^totaled, individual criterion fulfilled designated above)	N/A	271 (79.00)	271 (79.00)
Third-degree relative who has breast cancer and/or ovarian carcinoma and who has > 2 close blood relatives with breast cancer (at least one with breast cancer < 50 y) and/or ovarian carcinoma	N/A	2 (0.58)	2 (0.52)
*Total BRCA-related Criterion*	*70 (20.40)*	*273 (79.59)*	*343 (89.79)*
Lynch Syndrome (LS) NCCN Criteria (V2.2017)^f^ Fulfilled	Affected	Unaffected	Total Across Events
(*n* = 39)	n (% of LS Criteria met)		*N* = 382 (%)
Known Lynch syndrome mutation in the family	0 (0)	1 (2.56)	1 (0.26)
Family history of > 1 first-degree relative with colorectal or endometrial cancer diagnosed < 50 y	2 (5.19)	5 (12.82)	7 (.83).
Family history of > 1 first-degree relative diagnosed with colorectal or endometrial cancer and another synchronous or metachronous LS-related^e^ cancer	0 (0)	0 (0)	0 (0)
Family history of > 2 first-degree or second-degree relatives with LS-related cancer, including one diagnosed < 50 y	2 (5.13)	15 (38.46)	17 (4.45)
Family history of > 3 first-degree or second-degree relatives with LS-related cancer, regardless of age	0 (0)	11 (28.21)	11 (2.88)
Family meets Amsterdam II Criteria	0 (0)	3 (3.79)	3 (0.78)
*Total LS-related Criterion*	*4 (10.23)*	*35 (89.74)*	*39 (10.21)*
Total across all criteria (*N* = 382)	74 (19.37)	308 (80.63)	382 (100.00)

^a^includes invasive and ductal carcinoma in situ cancers; ^b^two breast cancer primaries included bilateral disease or two or more clearly separate ipsilateral primary tumor; ^c^close blood relatives include first-, and third-degree relatives on the same side of the family; ^d^includes fallopian tube and primary peritoneal cancers; ^e^LS-related cancers include colorectal, endometrial, gastric, ovarian, pancreas, ureter and renal pelvis, brain, small intestinal cancers, as well as sebaceous adenomas, sebaceous carcinomas, and keratoacanthomas as seen in Muir-Torre Syndrome; (^f^) no criteria with personal history fulfilled by cohort; specific criterion not reported in table above; ^g^“affected” has personal history of cancer; ^h^“unaffected” does not have personal history of cancer. Per NCCN guidelines

382/382 (100%) of events identified as fulfilling NCCN criteria by CGC pedigree review were also identified by the digital ID tool as fulfilling NCCN criteria. 343/382 (89.79%) independent events fulfilled BRCA-related criteria, 39/382 (10.21%) fulfilled Lynch syndrome-related criteria, and no histories fulfilled criteria for polyposis conditions (Table 3). The most common reasons for fulfilling NCCN BRCA-related genetic testing criteria were personal/family history of breast cancer diagnosed at or under 45 years of age, two breast cancers in the family with one diagnosed at or under 50 years of age, and the presence of ovarian cancer (Table 3). No personal history criteria were fulfilled for Lynch syndrome-related NCCN genetic testing; rather, all events fulfilled were related only to family history criteria.

### Low risk group

One hundred twenty-three pedigrees were included in the low risk group with 383 independent events recorded to assess against NCCN guidelines in CGC pedigree review and for entry into the digital ID tool, representing an event rate of approximately 3.11 events per pedigree. The majority of the low risk pedigrees (*n* = 120) were modeled by removing qualifying events from high-risk pedigrees; a small number (*n* = 3) were low-risk pedigrees residing in billing records, originally generated to fulfill pre-authorization requirements.

381/383 (99.47%) of events identified as not fulfilling NCCN criteria by CGC pedigree review were also identified by the digital ID tool as not fulfilling NCCN criteria (Table 4). Independent events of various tumor types were represented across degrees of relation with tumor types tested in 72 first-degree relatives (FDR), 195 s-degree relatives (SDR), and 14 third-degree relatives (TDR). The most frequently tested cancer types in the low risk group included female breast, colon, lung, prostate, melanoma and skin cancers (Table 4).

**Table 4:** Cancer types, degree of relation tested in low risk group events

Cancer Type	Degree of Relation^a^				Total
	FDR	SDR	TDR	Self	N (%)
Bladder	1	4	0	0	5 (1.30)
Bone	1	2	1	0	4 (1.04)
Breast	9	33	2	3	47 (12.30)
Cervical	2	4	1	3	10 (2.61)
CNS (Brain)	1	7	1	0	9 (2.35)
Colon - Cancer	5	18	4	0	27 (7.04)
Colon - Polyps	1	0	0	0	1 (0.26)
Gallbladder	0	1	0	0	1 (0.26)
Leukemia	3	9	1	0	13 (3.39)
Liver	0	2	0	0	2 (0.52)
Lung	11	23	0	0	34 (8.87)
Lymphoma - Unspecified	2	3	0	0	5 (1.30)
Lymphoma – Hodgkin’s	0	0	1	0	1 (0.26)
Lymphoma – Non-Hodgkin’s	1	4	0	1	6 (1.56)
Melanoma	7	9	1	4	21 (5.5)
Other	3	14	0	0	17 (4.43)
Pancreatic	4	10	0	0	14 (3.66)
Prostate	6	17	0	0	23 (6.00)
Renal	3	4	0	0	7 (1.82)
Sarcoma	1	0	0	0	1 (0.26)
Skin - Unspecified	6	16	1	3	26 (6.79)
Stomach	2	6	0	0	8 (2.09)
Thyroid	2	6	0	3	11 (2.87)
Uterine	1	3	1	1	6 (1.56)
No Cancer Family history	*–*	*–*	*–*	*–*	28 (7.31)
Combination histories	*–*	*–*	*–*	*–*	56 (14.6)
Total (n, %)	72 (18.80)	195 (50.91)	14 (3.66)	18 (4.70)	383 (100)

(^a^) Where “FDR” indicated first-degree Relative, “SDR” indicates second-degree relative, and “TDR” indicates third-degree relative

### Accuracy of the digital ID tool compared to pedigree review

True positive, true negative, false positive and false negative rates are reflected in Table 5. False positive events (i.e., low risk events identified as fulfilling NCCN criteria) included one pedigree describing a family history of brain cancer in a first-degree relative diagnosed at 45 years of age, and one (separate) pedigree describing brain cancer in a second-degree relative at 43 years of age.

**Table 5:** Accuracy calculations of digital id tool

	Presence of disease		
	Disease Present *(Meets NCCN Criteria on CGC Review)*	Disease Absent *(Does not meet NCCN Criteria on CGC Review)*	Total
Test outcome			
Positive *(Meets NCCN Criteria by digital ID tool)*	382 (A)	2 (B)	384
Negative *(Does not meet NCCN criteria on digital ID tool)*	0 (C)	381 (D)	381
Total	382	383	765
	*Formula Used*	*Calculation*	*Value* ^a^
*Sensitivity*	A/(A + C)	382/382	100%
*Specificity*	D/(D + B)	381/383	99.48%
*Accuracy*	(A + D)/(A + B + C + D)	763/765	99.74%

^a^Values confirmed to be within 95% confidence intervals using related statistical methods and formulas

The digital ID tool was calculated to have a sensitivity of 100% (99.04–100% CI) and specificity of 99.48% (98.13–99.94% CI). The overall accuracy of the digital ID tool is estimated to be 99.74% (99.06–99.97% CI), reflecting the rate at which the digital ID tool reached the same conclusion as CGC three-generation pedigree review.

## Discussion

This study describes the development and validation of a digital ID tool that automatically and accurately identifies individuals at risk for common HCS, according to NCCN criteria. The high sensitivity and specificity of this tool suggests it may be useful in clinical practice, specifically for BRCA-related HBOC and Lynch syndrome. The digital ID tool provides a complement to patient preferences, provider clinical experience and intuition, and standards of best practice that comprise clinical recommendations to offer genetic screening for hereditary cancer syndromes.

Data analyzed in this study provide unique insight into the use of a digital ID tool for a largely unstudied at-risk unaffected population. The United States Preventive Services Task Force identifies five screening tools with “high” sensitivity of > 85% for use of identification of unaffected, at-risk individuals for BRCA-related testing [15]. With > 99% sensitivity and specificity, the digital ID tool validated in this study appears to be a more accurate mechanism for identifying at-risk individuals than other broad screens or digital solutions currently available for hereditary cancer risk assessment [17–19]. However, accuracy of this tool was tested using a clinical genetics expert; accuracy of it remains to be tested when used in clinical settings by non-genetics providers.

The digital ID tool may offer an impactful solution to address current gaps observed in BRCA-related genetic counseling and testing settings [6, 8, 9]. BRCA-related risk criteria recorded most frequently during pedigree review in this study, including personal and/or family history of breast cancer before 45 years of age, two or more breast cancers including one diagnosed under 50 years of age, and ovarian cancer at any age, may represent both the most common reasons for referral for genetic testing and criteria that are most familiar to health care providers. Studies have estimated that these criteria alone may identify nearly one million eligible women for hereditary cancer screening [6], supporting the potential impact of clinical integration of a valid tool to facilitate identification of at-risk individuals not currently identified.

The lower number of Lynch syndrome tested events in this study may be reflective of gaps in knowledge and awareness of Lynch syndrome by ordering providers and low rates of Lynch syndrome identification [7, 10]. While validated across NCCN criteria, the difference in the volume of tested events in this study for Lynch syndrome compared to those for BRCA-related HBOC is striking. Such trends are not unique to this study as others have noted significantly higher rates of genetic referral and testing for breast cancer than for colon cancer as well as lower rates of family history documentation for patients with colon cancer compared to those with breast cancer [11]. However, family history collection with tools similar to the digital ID tool have been validated in the Lynch syndrome population [17], suggesting that the digital ID tool is also likely useful in this population.

No polyposis events were captured in CGC pedigree review or tested by the digital ID tool due to lack of presence of cases in the randomized cohort. Therefore, we cannot make conclusions about the validity of the digital ID tool in a population at risk for polyposis syndromes. Additional studies evaluating patients in a gastroenterology setting may be needed to test validity of the digital ID study for polyposis syndromes.

The modeled cases included in the low risk group are representative of broad populations that would be assessed as low risk by the digital ID tool in non-modeled cases as analyzed cancer histories are similar in distribution to those of the general population [28]. Knowledge and accuracy of family history decrease with degree of relation [29–31], so it was not surprising that information about first- and second-degree relatives was more frequent in tested events than information about third-degree relatives. Recommendations for the collection of family history for both affected and unaffected populations suggest that inclusion of first- and second-degree relatives from both lineages is sufficient for risk assessment [13, 32]; by extension, such histories may also be sufficient for identification of at-risk individuals for HCS by clinically valid tools, including the digital ID tool.

Limitations exist in this study. The study design was retrospective in nature. The majority of low risk cases were modeled since they are challenging to otherwise ascertain. The study cohort was all female and largely comprised of Caucasians without a personal history of cancer, which may reduce its generalizability to other populations. Also, the digital ID tool is intended for patient-facing entry of information; in this study, a CGC entered history from pedigrees into the digital ID tool. Further study is needed to determine the accuracy of the tool when used by patients in the clinical setting. A prospective study is already underway by our laboratory to better understand how patients record their personal and/or family cancer histories into the digital ID tool compared to information they provide to a CGC, patient experiences using the tool, and the accuracy of the tool’s output when interpreted by a non-genetics provider.

Lastly, the ability of the digital ID tool to identify individuals at risk for HCS is also affected by limitations in the NCCN guidelines. Studies evaluating multi-gene cancer panel use have suggested that NCCN criteria may not capture all patients at risk for HCS [33–36]. A digital ID tool could employ expanded criteria to identify such patients, however further studies should continue to examine the sensitivity of the NCCN criteria as well as the costs and benefits of expanding NCCN criteria prior to integration into a valid tool.

## Conclusions

The digital ID tool has high sensitivity and specificity, and with further testing in the clinical setting, could be used for accurate, automated identification of individuals at risk for common HCS. The digital ID tool could serve as an alternative to provider-mediated pedigree collection as a means of collecting family history, as well as aid in the determination of whether genetic testing for HCS is needed. By expediting family history collection in a clinically valid manner, strides may be made in closing the gap for the large numbers of at-risk individuals currently lacking knowledge of their hereditary cancer risks.

## Additional file

Additional file 1: **Figure S1.** Selected screenshots of Digital ID Tool as viewed on mobile device (as of February 2018). (PDF 2668 kb)

## Abbreviations

FDR: First-degree relative
HBOC: Hereditary Breast and Ovarian Cancer
HCS: Hereditary Cancer Syndromes
NCCN: National Comprehensive Cancer Network
SDR: Second-degree relative
TDR: Third-degree relative

## References

[1] Garber JE, Offit K (2005). Hereditary cancer predisposition syndromes. J Clin Oncol.

[2] Coughlin SS, Khoury MJ, Steinberg KK (1999). BRCA1 and BRCA2 gene mutations and risk of breast cancer: public health perspectives. Am J Prev Med.

[3] Hampel H, de la Chapelle A (2013). How do we approach the goal of identifying everyone with lynch syndrome?. Familial Cancer.

[4] NCCN Clinical Practice in Oncology (NCCN Guidelines). Genetic/Familial High-Risk Assessment: Breast and Ovarian. https://www.nccn.org/professionals/physician_gls/pdf/genetics_screening.pdf Accessed 25 Jan 2018.

[5] NCCN Clinical Practice in Oncology (NCCN Guidelines). Genetic/Familial High-Risk Assessment: Colorectal.https://www.nccn.org/professionals/physician_gls/pdf/genetics_colon.pdf Accessed 25 Jan 2018.

[6] Childers CP, Childers KK, Maggard-Gibbons M, Macinko J. National estimates of genetic testing in women with a history of breast or ovarian cancer. J Clin Oncol. 2017;doi: 10.1200/JCO.2017.73.6314. 10.1200/JCO.2017.73.6314 PMC5707208 28820644

[7] Patel SG, Ahnen DJ, Kinney AY (2016). (2016). Knowledge and uptake of genetic counseling and colonoscopic screening among individuals at increased risk for lynch syndrome and their endoscopists from the family health promotion project. Am J Gastroenterol.

[8] Bellcross CA, Peipins LA, McCarty FA, Rodriguez JL (2015). Characteristics associated with genetic counseling referral and BRCA1/2 testing among women in a large integrated health system. Genet Med..

[9] Hull LE, Haas SE, Simon SR (2018). Provider discussions of genetic tests with U.S. women at risk for a BRCA mutation. Am J Prev Med.

[10] Drohan B, Roche CA, Cusack JC, Hughes KS (2012). Hereditary breast and ovarian cancer syndromes: using technology to identify carriers. Ann Surg Oncol.

[11] Wood ME, Kulback P, Pham TH, Wollins DS (2014). Quality of cancer family history and referral for genetic counseling and testing among oncology practices: a pilot test of quality measures as part of the American Society of Clinical Oncology quality oncology practice initiative. J Clin Oncol.

[12] Diamonstein C, Stevens B, Shahkruh Hashmi S, Refuerzo J, et al. Physicians' awareness and utilization of genetic Services in Texas. J Genet Couns. 2017. 10.1007/s10897-017-0199-z. 10.1007/s10897-017-0199-z 29280038

[13] Lu KH, Wood ME, Daniels M, Burke C (2014). American Society of Clinical Oncology expert statement: collection and use of a cancer family history for oncology providers. J Clin Oncol.

[14] Vig HS, Armstrong J, Egleston BL, Mazar C (2009). Cancer genetic risk assessment and referral patterns in primary care. Genet Test Mol Biomarkers.

[15] United States Preventive Services Task Force. Final Recommendation Statement on BRCA-Related Cancer: Risk Assessment, Genetic Counseling, and Genetic Testing. 2018. https://www.uspreventiveservicestaskforce.org/Page/Document/UpdateSummaryFinal/brca-related-cancer-risk-assessment-genetic-counseling-and-genetic-testing. Accessed 25 Jan 2018.

[16] Taxler BL, Martin ML, Kerber AS, Bellcross CA (2014). Implementation of a screening tool for identifying patients at risk for hereditary breast and ovarian cancer: a statewide initiative. Ann Surg Oncol.

[17] Kallenberg FG, IJspeert JE, Bossuyt PM, Aalfs CM, Dekker E (2015). Validation of an online questionnaire for identifying people at risk of familial and hereditary colorectal cancer. Familial Cancer.

[18] Acheson LS, Zyzanski S, Stange KC (2006). Validation of a self-administered, computerized tool for collecting and displaying the family history of cancer. J Clin Oncol.

[19] Wu RR, Himmel TL, Buchanan AH, Powell KP (2014). Quality of family history collection with use of a patient facing family history assessment tool. BMC Fam Pract.

[20] Wang C, Bickmore T, Bowen DJ (2015). Acceptability and feasibility of a virtual counselor (VICKY) to collect family health histories. Genet Med.

[21] Qureshi N, Wilson B, Santaguida P, Carroll J, et al. Collection and use of cancer family history in primary care. Evid Rep Technol Assess (Full Rep). 2007;(159):1–84. PMC4781030 18457477

[22] Pritzlaff M, Yorczyk A, Robinson LS, Pirzadeh-Miller S (2014). An internal performance assessment of CancerGene Connect: an electronic tool to streamline, measure and improve the genetic counseling process. J Genet Couns.

[23] Birch PH (2015). Interactive e­counselling for genetics pre­test decisions: where are we now?. Clin Genet.

[24] Doerr M, Edelman E, Gabitzsch E (2014). Formative evaluation of clinician experience with integrating family history­-based clinical decision support into clinical practice. J Pers Med.

[25] Kang HH, Williams R, Leary J (2006). Evaluation of models to predict BRCA mutations. Br J Cancer.

[26] Kastrinos F, Steyerberg EW, Mercado R, Balmaña J (2011). The PREMM(1,2,6) model predicts risk of MLH1, MSH2, and MSH6 germline mutations based on cancer history. Gastroenterology.

[27] Panchal SM, Ennis M, Canon S, Bordeleau LJ (2008). Selecting a BRCA risk assessment model for use in a familial cancer clinic. BMC Med Genet.

[28] American Cancer Society. Cancer facts and figures 2017. https://www.cancer.org/content/dam/cancer-org/research/cancer-facts-and-statistics/annual-cancer-facts-and-figures/2017/cancer-facts-and-figures-2017.pdf. Accessed 25 Jan 2018.

[29] Ozanne EM, O’Connell A, Bouzan C, Bosinoff P (2012). Bias in the reporting of family history: implications for clinical care. J Genet Couns.

[30] Gaff CL, Aragona C, MacInnis RJ, Cowan R (2004). Accuracy and completeness in reporting family history of prostate cancer by unaffected men. Urology.

[31] Ziogas A, Anton-Culver H (2003). Validation of family history data in cancer family registries. Am J Prev Med.

[32] Modesitt SC, Lu K, Chen L, Powell CB (2017). Of the committee on practice bulletins–gynecology, committee on genetics, Society of Gynecologic Oncology. Practice bulletin no 182: hereditary breast and ovarian Cancer syndrome. Obstet Gynecol.

[33] LaDuca H, Stuenkel AJ, Dolinsky JS, Keiles S (2014). Utilization of multigene panels in hereditary cancer predisposition testing: analysis of more than 2,000 patients. Genet Med.

[34] Couch FJ, Hart SN, Sharma P, Toland AE (2015). Inherited mutations in 17 breast cancer susceptibility genes among a large triple-negative breast cancer cohort unselected for family history of breast cancer. J Clin Oncol.

[35] Frey MK, Sandler G, Sobolev R, Kim SH (2017). Multigene panels in Ashkenazi Jewish patients yield high rates of actionable mutations in multiple non-BRCA cancer-associated genes. Gynecol Oncol.

[36] O’Leary E, Iacoboni D, Holle J, Michalski ST (2017). Expanded gene panel use for women with breast Cancer: identification and intervention beyond breast Cancer risk. Ann Surg Oncol.

