# Supplementary material for: Validation of a digital identification tool for individuals at risk for hereditary cancer syndromes
Source: Hered Cancer Clin Pract. 2019 Jan 11;17:2. doi: 10.1186/s13053-018-0099-8 (PMC6330430; doi:10.1186/s13053-018-0099-8)

Supplemental Figure 1. Selected screenshots of Digital ID Tool as viewed on mobile device
**As of February 2018*

Personal History Questions

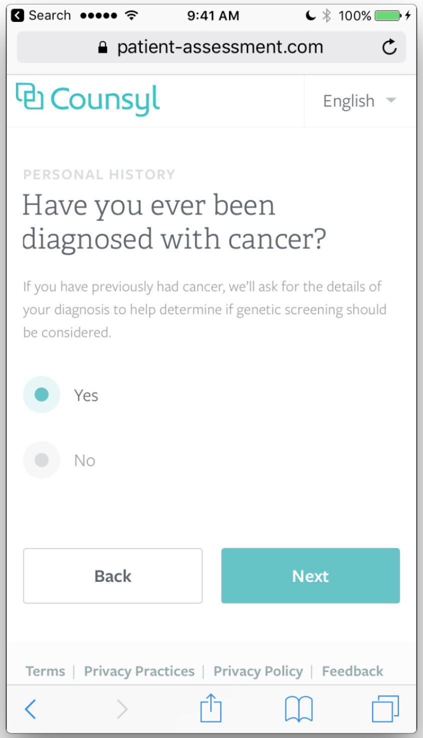

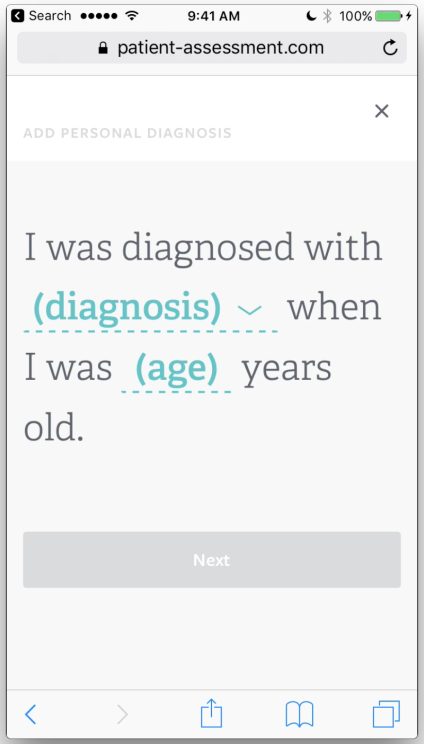

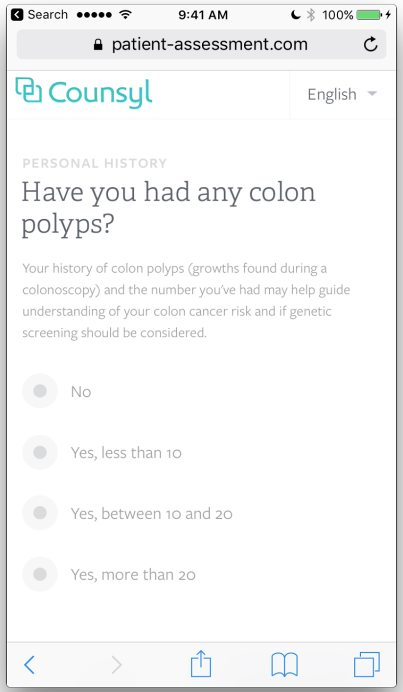

Family History Questions

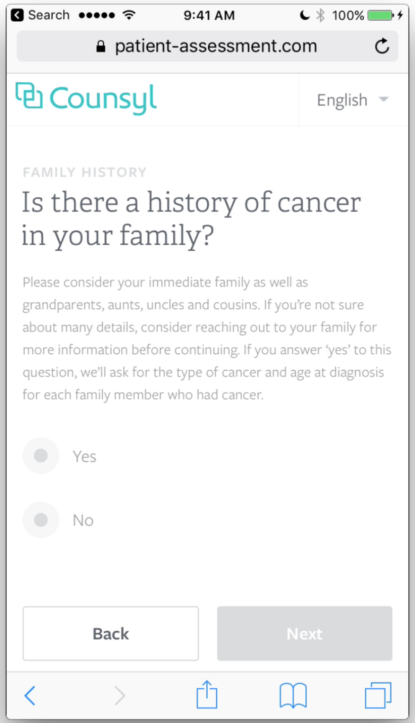

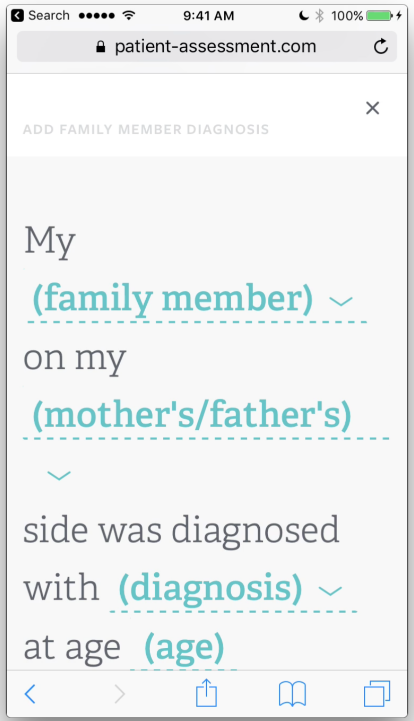

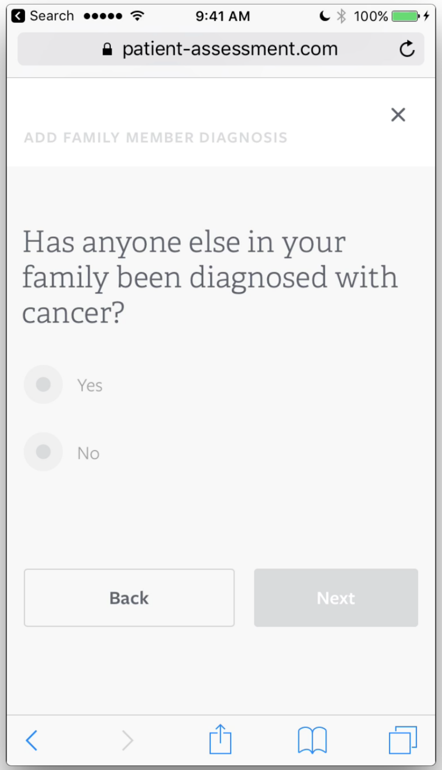

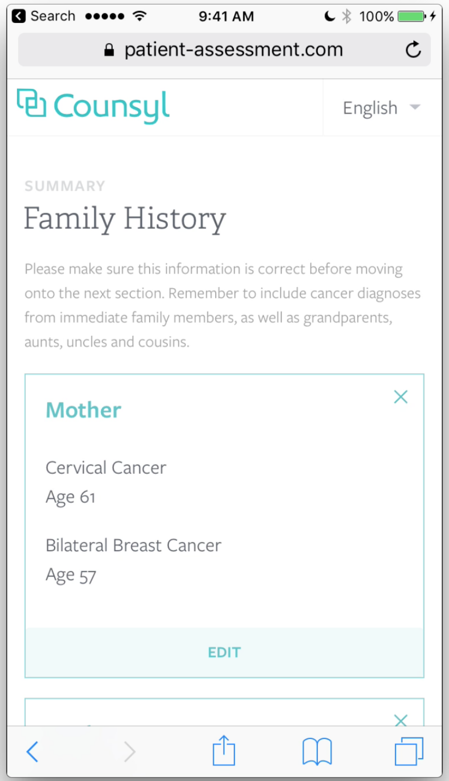

Additional Questions and Review

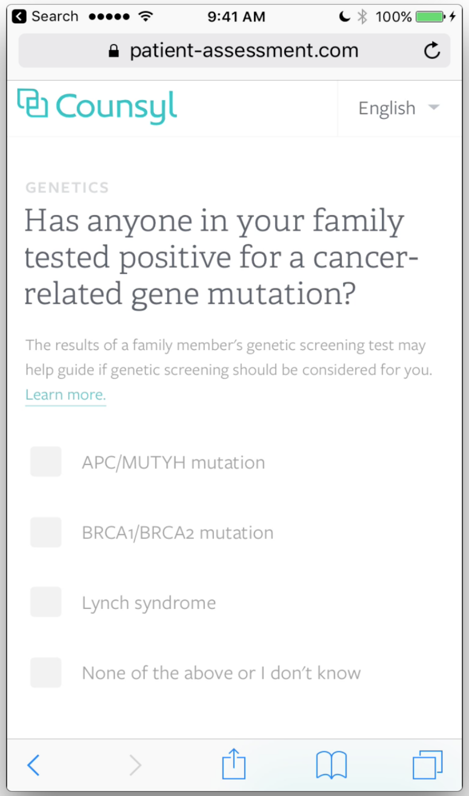

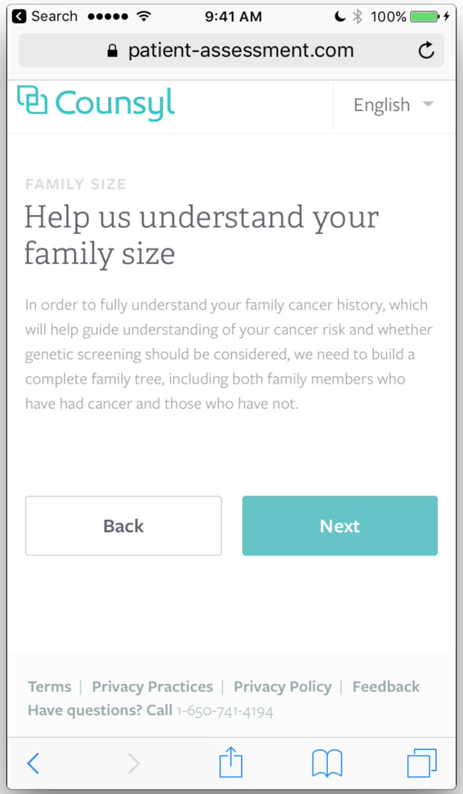

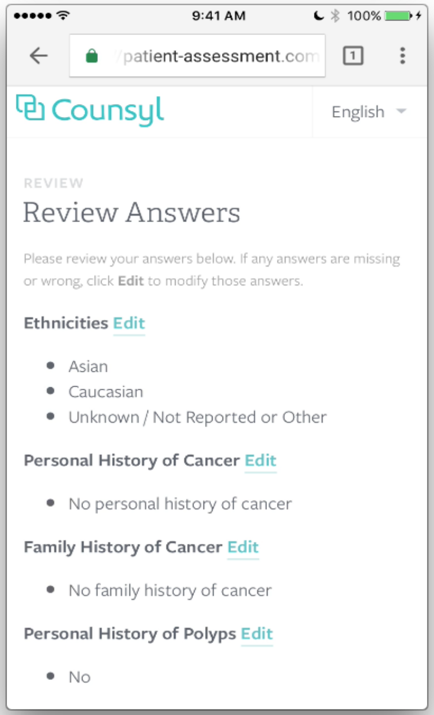

Supplement: Supplementary file 1 — Figure S1. Selected screenshots of Digital ID Tool as viewed on mobile device (as of February 2018). (PDF 2668 kb) [file 13053_2018_99_MOESM1_ESM.pdf]
